# Recent data on cannabinoids and their pharmacological implications in neuropathic pain

**Published:** 2008-11-15

**Authors:** Andreia Coman Oana, Paunescu Horia, Coman Laurentiu, Badarau Anca, Fulga Ion

**Affiliations:** * „Carol Davila” University of Medicine and Pharmacy, Bucharest, Romania

**Keywords:** neuropathic pain, cannabinoids, analgesia

## Abstract

Natural cannabinoids have been used for centuries for their psychotropic properties, but their possible therapeutic implications in analgesia have been recently documented. The present review intended to make an analysis of the neuroanatomy and physiology of the cannabinoid system (receptors, functions, agents acting on these receptors) and of its implications in neuropathic pain. There were also described the complex phenomena implicated in the generation and maintenance of neuropathic pain, by high lightening the implications of endogenous cannabinoids in this complex of painful conditions. The pharmacological analgesia test proves of cannabinoid implication in neuropathic pain was sustained by many studies presented in this paper. Therapeutic approaches using natural and synthetic cannabinoid receptor agonists were reviewed. Therapeutic perspectives in neuropathic pain might involve the development of new agents that influence the cannabinoid system. Thus, peripheral acting cannabinoid 1 receptors agonists, selective cannabinoid 2 receptor agonists and also modulators of endocannabinoids metabolism might be a way to success in the treatment of this complex entity called neuropathic pain.

## Introduction-history and summary of cannabinoids effects 

Although psychotropic properties and some therapeutic actions of natural cannabinoids (extracted from *Cannabis sativa*) are well known and documented since thousands of years, it was only in 1964 when Ganoi and Mechoulam identified ∆9 tetrahydrocannabinol, being the main psychotropic agent from this plant [**1**].

The benefic therapeutic effects of cannabinoids could be: analgesia, attenuation of nausea and vomiting in cancer chemotherapy, reduction of intraocular pressure, appetite stimulation in wasting syndromes, relief from muscle spasms/spasticity in multiple sclerosis and decreased intestinal motility. But there are also produced adverse reactions like: alterations in cognition and memory, dysphoria/euphoria, and sedation. Animal models that could evoke cannabinoid receptor activity include drug discrimination paradigms in rodents, pigeons and nonhuman primates, a typical static ataxia in dogs and a tetrad of responses in rodents (hypothermia, analgesia, hypo activity and catalepsy) [**2**].

## Cannabinoid receptors (CBR) – history, characterization, localization, functions

Initially, it was believed that these compounds act unspecifically, by influencing the neuronal membrane and this hypothesis seemed believable because of the high liposolubility of Δ9 tetrahydrocannabinol (THC) and related compounds. Lawrence & Gill proposed in 1975 that „it is unnecessary to invoke the existence of a specific cannabinoid receptor” and to propose that the psycho activity of cannabinoids results from a structure-dependent ability to disorder membrane lipids [**3**].

But in the late eighties appeared some proves of the existence of some receptors for cannabinoids, proves appeared after using radioligands. The existence of some satiable bounding sites, stereo selective and with high affinity for cannabinoids, located on the mammals’ brain, was reported [**4**]. Afterwards, the first type of cannabinoid receptor was cloned out of a library of rat complementary DNA and was named CB1 [**5**]. The second type of cannabinoid receptor, CB2, was identified for the first time on a human promielocitary leukemia cells line [**6**]. After that, there were described ligands of cannabinoid receptors with a different structure than that of natural cannabinoids. Some of these structures were endogenous molecules and were named endocannabinoids. The most important endocannabinoid is probably anandamide, or arachidonoylethanolamide, isolated from water insoluble fractions from the pig brain. The name of anandamide came from Sanskrit, where ananda signified supreme happiness, bliss [**7**].

The human CB1 receptor (hCB1) cloned by Gerrard et al. (1991) [**8**] had a 97% homology in the sequence of amino acids with the mouse or rat CB1 receptor. Two splice variants of the CB1 receptor (CB1a and CB1b) were identified, but they had smaller tissular diffusion than the initial variant (full length) [**9**].

CB1 receptors are coupled with Gi/Go proteins and are serpentine receptors with the N-terminal end extracellular and the C-terminal end intracellular. The C-terminal end is implicated in the signal transmission to G proteins, controls the recirculation by internalization after ligand binding [**10**] and desensitization after prolonged exposition to an agonist (more than 2 hours) [**11**].

The proximal segment of the C-terminal end of the receptor is implicated in bounding of G proteins [**12**]. Through G protein action the activity of adenylyl-cyclase is diminished which leads to a decrease of cAMP level. Also through some G proteins action the activity of some ionic channels is modulated. CB1 receptors are positively coupled with potassium channels type A, M and inwardly rectifying potassium channels and negatively coupled with type L, N and P/Q of voltage dependent calcium channels and also with type D potassium channels. CB1 receptors could also activate phospholipase A through G proteins that contain Gα14, Gα15 and Gα16 subunits [**13**].

A part of the CB1 receptors is found at the nervous presynaptic endings, the effect of their activation being the inhibition of synaptic transmission, especially the GABA-ergic and glutamatergic one.

In the central nervous system, cannabinoid receptors type CB1 are found in the cerebral cortex, hippocampus, caudate-putamen, substantia nigra pars reticulata, globus pallidus, entopeduncular nucleus and cerebellum [**13**].

CB2 receptors were first identified in men in 1993 and then in rats and mice. The animal receptor had a 81-82% structural homology with the CB2 human receptors. [**6**] Unlike CB1 receptors, the CB2 ones didn’t seem to be coupled with Go type proteins or with ionic channels. They are coupled with intracellular signalization pathways associated to MAP kinase and to B-protein kinase [**14**].

CB2 receptors are found especially on the immune system cells. The presence of the CB2 receptors has been proved on the microglia cells and in the cerebellum in man [**15**]. There are some studies that did not showed the presence of CB2 receptors on the neurons in men and rats [**6**].

Another two serpentine receptors, being classified among orphan receptors because when discovered there did not exist a specific ligand to bind them, were supposed to be cannabinoid receptors. These two receptors are still named GPR55 and GPR119 [**16**].

Endogenous ligands for CB receptors discovered until now are eicosanoids: N-arachidonoylethanolamide (anandamide), 2-arachidonoyl glycerol, noladin ether, O-arachidonoylethanolamine (virodhamide) and N-arachidonoyldopamine.

A short presentation of endocannabinoids metabolism is useful for understanding the ways to influence these pathways. Three of the 5 putative endocannabinoids - anandamide, 2-arachidonoyl glycerol, and N-arachidonoyldopamine - are susceptible to degradation by fatty acid amide hydrolase (FAAH), although a second enzyme, monoacylglycerol lipase (MGL), catalyzes hydrolysis of 2--arachidonoylglycerol *in vivo*.

Cellular uptake of anandamide reportedly involves facilitated diffusion, although a specific transporter has yet to be cloned. Kinetics studies suggest the presence of an anandamide membrane transporter, and pharmacological studies using inhibitors of anandamide transport have supported the notion that anandamide transport inhibition has a role in modulating endocannabinoid tone. Among the most commonly employed drugs of this class is AM404 [**17**].

## Agonists, antagonists/inverse agonists of cannabinoid receptors

Nowadays, there are described more than 60 substances acting on the endogenous cannabinoid system. Their classification depending on their chemical structure, in accord with IUPHAR (International Union of Pharmacology), is presented in [**Table nr.1**.

**Table 1:** Substances acting on the endogenous cannabinoid system.

Cannabinoid receptor agonists		
Classical cannabinoids	Δ9 THC	partial agonist of CB1R and CB2R
Non-classical cannabinoids	CP-55,940	complete agonist of both CB1R and CB2R
Specific CB-2 receptor agonist	AM 1241	
Aminoalkylindoles	R-(+)-WIN-55,212-2	complete agonist of both CB1R and CB2R, slighty selective for CB2R
Eicosanoids	anandamide	partial agonist of both CB1R and CB2R and TRPV1 agonist*
	R-(+)-methanandamide arachidonoyl- 2’-chloroethylamide (ACEA).	
Cannabinoid receptor antagonists/inverse agonists**		
Diarylpyrazoles	SR141716A [rimonabant]	selective CB1R blocker
	SR144528	selective CB2R blocker
Substituted benzofuranes	LY 320135	CB1, 5-HT, muscarinic receptors
Aminoalkylidoles	AM 630	CB2R antagonist, partial CB1R agonist
Triazole derivatives	LH-21	CB1R antagonist
Uptake blockers		
AM 404, UCM 707, AM1172		
Carbamate FAAH inhibitors		
OL-135, URB 597, URB 532		
* Anandamide is also described as agonist of transient receptor potential vanilloid1 (TRPV1) receptors, receptors pharmacologically activated by capsaicin [**17**].		
**The notion of inverse agonist rests on the ability of the CB1 and CB2 receptors to exhibit signal transduction activity in the absence of endogenous or exogenous agonists (constitutive activity). As such, arylpyrazoles can behave as “inverse agonists” in order to reduce the constitutive activity of the signal transduction pathways [**2**].		
CB1R – CB1 receptor		
CB2R – CB2 receptor		

Δ9-THC, CP-55,940, WIN-55,212 and many of their analogues contain chiral centers and exhibit marked stereoselectivity in both binding assays and functional tests. For classical and non-classical cannabinoids those with stereochemistry like (-)-Δ9-THC have the greater activity. For example, the non-classical cannabinoid CP-55,940 is a (-)-enantiomer and has higher affinity for CB1 or CB2 receptors than its (+)-enantiomer. Contrarily the (+) enantiomer of WIN-55,212 is more active.

Stereoselectivity of action, besides dose-analgesic effect relationship, blockade of agonists effects by substances found out to be antagonists are part of the proves for their action on specific receptors (identified to be CB1 and CB2) [**13**].

GPR55 binds to and is activated by the cannabinoid ligand CP-55,940. In addition endocannabinoids, including anandamide and virodhamine, activate GTPγS binding via GPR55 with nanomolar potencies. Ligands such as cannabidiol and abnormal cannabidiol (extracted from cannabis) which exhibit no CB1 or CB2 activity and are believed to function at a novel cannabinoid receptor, also showed activity on GPR55. Thus, GPR55, described as an orphan receptor, could be the third cannabinoid receptor. Another orphan receptor GPR 119, is activated by oleoylethanolamide, a substance close related to eicosanoids, and seems to be the forth cannabinoid receptor [**16**].

## Neuropathic pain – definition, histopathological changes connected to chronic (neuropathic) pain

Neuropathic or neurogenic pain means a complex of painful states which takes place in the nervous sensorial axis which connects the nervous sensorial endings with cortical areas. The most frequently this sensation comes from the sensorial peripheral nerves and from the posterior medullar horns [**18**].

This type of sensation is determined by injuries of the nervous tissue others than those implicated in the simple nociception. This simple nociception is referring to injuries that don’t produce the morphological destruction of nociceptors. There are also borderline states in which pain has different components: one is given by lesions of the peripheral or central nervous system and one is due to the residual implication of nociceptors at the site of the initial tissular injury generating a mixt nociceptive-neuropathic pattern [**19**].

Even if there are a great variety of causes implicated in the etiology of neuropathic pain, its symptoms are dominated by the dichotomy between positive and negative symptoms which correlates to the anatomic distribution of the damaged nerve. Negative symptoms include total or partial loss of sensibility, atrophy and muscular weakness. Positive symptoms include static mechanic allodynia, paresthesia, dysesthesia, and thermal hyperalgesia, painful sensations after the end of the stimulus, pain without stimuli (spontaneous), symptoms that generate continuous or intermittent paroxysmal pain [**20**].

Allodynia, Latin name that signifies „another pain”, is an exaggerated answer (reaction) to stimuli that normally don’t produce pain. There are described: static mechanic allodynia – pain as an answer to light touch or pressure, dynamic mechanic allodynia – pain as an answer to „brushing” (touch with a cotton wool) [**21**]

Hyperalgesia suppose exaggerated pain to a stimuli that normally don’t induce pain.[**19**]

The spectrum of neuropathic pain covers a variety of disease states and has a variety of symptoms. Several etiologies of peripheral nerve injury might result in neuropathic pain: post herpetic neuralgia, traumatic injury, phantom limb pain, diabetes and malignancy.

In chronic pain, an important mechanism in the generation of painful sensations is represented by the *peripheral sensitization* of sensorial neurons. They pester with nervous impulses the spinal cord leading to an excitability increase and to synaptic damages in the dorsal horns of the spinal cord. This phenomenon is called *central sensitization*.

At the cellular level, a very important role in these sensitization processes is hold by the synaptic transmission and by the translation of the peripheral signal to the nervous cells implicated in nociception [**22**].

Peripheral sensitization

Anatomic and physiologic changes in peripheral nerves can induce pain. After an axon is cut, the part still connected to the nerve cell body seals and forms an end bulb (terminal swelling). Dying back of the axon may occur with an associated disruption of the myelin sheath, or one or more axonal sprouts may emerge from the end bulbs and elongate. When sprouts reach peripheral target tissue, peripheral receptor activity restarts and growth ends. 

Even if the majority of peripheral nerve function is restored, some sprouts may become trapped somewhere along the nerve and form „neuroma in continuity.” If a nerve is only partially transected, axonal regrowth may develop into multiple microneuromas.

Impulses may occur secondary to multiple depolarizing stimuli: pressure, temperature changes, ischemia, changes in blood oxygen levels, increased extracellular potassium concentration and the effects of various peptides and neurotransmitters.

Neuromas are associated with the induction of chronic pain in the peripheral nerves associated with the stumps of amputated limbs, including phantom limb pain.

Areas of demyelination in otherwise normal peripheral nerves may be associated with the generation of ectopic impulses. Pain from such areas is associated with electrical-like or lancinating pain [**19**].

Central sensitization

Three types of central changes are hypothesized.

1. Changes in afferent impulses, which can induce long-term shifts in central synaptic excitability. 

2. Peripheral sensitization leads to changes in chemical substances from the periphery and this produces changes in spinal cord excitability [**23**]

3. Pathological hyperexcitability may be secondary to changes in central control mechanisms.

1. After a nerve or tissue injury, dorsal horn receptive fields may increase in size, decrease their action potential threshold and/or increase their responsiveness to nociceptive information in a way that exactly parallels changes in pain sensitivity. When peripheral nerve primary afferents are damaged, significant topographic reorganization of the primary afferent terminals in the spinal cord may occur. Thus, large myelinated mechanoceptive Aβ-axons sprout into lamina II, an area in which they do not normally terminate [**24**]. The functional importance of Aβ-fiber sprouting is that lamina II begins to receive information about non-noxious stimuli. This information may be misinterpreted by the CNS as noxious; this is an anatomical substrate for mechanical allodynia.

2. Abnormal hyperexcitability of central nociceptive neurons appears to be highly dependent on the activation of the N-methyl-D-aspartate (NMDA) glutamatergic receptors located on the membrane of spinal cord dorsal horn neurons secondary to massive release of excitatory amino acids (particularly glutamate and aspartate).

3. Central descending modulating systems can induce inhibition or facilitate excitability on the dorsal horn systems. Supposed opioid resistance in neuropathic pain may have several etiologies: a reduction of the inhibitory influence over spinal transmission after nerve injury as a result of decreased opioid receptors number on sensory neurons, a decrement in GABA content within the dorsal horn , and interneuron transsynaptic cell death following nerve injury. The spinal neuronal membrane is thus rendered hyperexcitable secondary to disinhibition [**19**].

## Neuroanathomic proves of CB receptors existence in neuropathic pain pathways 

In autoradiographic studies, high affinity specific binding sites for [3H]CP-55940 have been detected not only within the spinal cord but also in rat trigeminal ganglia, pointing to the presence of cannabinoid receptors on primary afferent neurons [**25**].

The dorsal root ganglion (DRG) has been used as a model of the peripheral nerve because of its more convenient size, location and the ability to correlate cell size and neurochemical phenotype with peripheral axon caliber. Two basic types are generally recognized in DRG: large, light A cells and small, dark B cells but some authors describe a third type: intermediate sized neurons. The largest A cells are the typical proprioceptor neurons and the small B cells are the typical nociceptor neurons [**26**].

Hohmann and Herkenham (1999) showed that dorsal root ganglion cells, the source of primary afferent input to the spinal cord, synthesize cannabinoid CB1Rs. CB1R mRNA was highly expressed in dorsal root ganglion cells of heterogeneous cell size, and predominant in intermediate-sized neurons [**27**]. Salio et al.2002 confirmed the presence of CB1Rs in small, intermediary and large cells of rat dorsal root ganglia [**28**].

Other observations indicate that under normal conditions, CB1Rs are localized mainly on non-nociceptive primary afferent fibers [**29**].

Contrary, a study revealed that in normal rats CB1R is expressed in the majority (76–83%) of nociceptive neurons [**30**].

The phenotypes of cells expressing CB1Rs in native DRG differs from that reported in cultured DRG, where co localization of CB1Rs with markers of nociceptors is more prevalent [**31**].

Both CB1R and CB2R have been identified in primary cultures of dorsal root ganglion cells derived from neonatal rats [**32**]. It is unclear if CB2Rs are expressed in satellite glial cells, the main glial cells in sensory ganglia that have been shown to be histological altered in animal models of nociception [**33**]. Neuronal expression of CB2R mRNA in native DRG [**34**] and trigeminal ganglia was similar to background under conditions in which CB1R mRNA was clearly demonstrated [**35**].

There was considerable support for postsynaptic localization of CBRs in rat spinal dorsal horn cord [**36**]. This pattern might suggest an anatomical basis for the efficacy of cannabinoids in ameliorating inflammatory and neuropathic pain [**37**].

Presynaptic receptors were evidenced in heterogeneous dorsal root ganglion neurons and in axons of Lissauer's tract. CB1R immunoreactivity had also been localized to dorsal horn interneurons containing γ-aminobutyric acid (GABA) [**38**].

Direct evidence for supraspinal sites of cannabinoid antinociception was derived from studies employing intracranial administration of cannabinoids. Site-specific injections of cannabinoid agonists to various brain regions have permitted the identification of brain loci implicated in cannabinoid antinociception. The active sites included the dorsolateral periaqueductal gray, dorsal raphe nucleus, rostroventromedial medulla, amygdala, lateral posterior and submedius regions of the thalamus, superior colliculus, and noradrenergic A5 region. These studies suggested that endocannabinoid actions at these sites were sufficient to produce antinociception [**39**, **40**, **41**].

At the level of the periaqueductal gray, metabotropic glutamate and N-methyl-d-aspartate (NMDA) receptors were required for cannabinoid antinociception [**42**].

*In vitro* studies demonstrated that cannabinoids inhibit GABA and glutamate release presynaptically in the periaqueductal gray in the absence of direct postsynaptic effects on periaqueductal gray neurons [**43**].

Researchers have targeted synthetic cannabinoids at other brainstem nuclei including the rostroventromedial medulla [**40**, **44**, **45**] and the nucleus reticularis gigantocellularis [**44**] to better characterize sites of cannabinoid-mediated antinociception. Site-specific administration of cannabinoids in the rostroventromedial medulla produced significant antinociception [**40**].

At the cellular level, it appeared that cannabinoids exert their physiological effects in the rostroventromedial medulla by presynaptic inhibition of GABAergic neurotransmission [**45**].

## Pharmacological analgesia test proves of cannabinoids implication in neuropathic pain

Some of the sensory abnormalities associated with neuropathic pain - allodynia and hyperalgesia - have been reproduced by injury to peripheral nerves in animal models. Cannabinoids treatment reduced some of the effects of neuropathic pain.

After performing *unilateral chronic constriction injury* (CCI) to the rat sciatic nerve, behavioral hypersensitivity to cold, mechanical and thermal stimuli can be measured by timing hind paw withdrawal latencies.

Herzberg et al. (1997) showed that systemic application of WIN-55,212-2, a CBR agonist, was effective in reducing heat and mechanical hyperalgesia and mechanical and cold allodynia following the induction of neuropathy. These findings were obtained at doses that did not affect withdrawal latencies of hind paws contra lateral to the injury and did not produce systemic effects [**46**].

Also Herzberg et al. (1997) obtained evidence that SR141716A, a selective CBR1 antagonist, enhanced thermal hyperalgesia and mechanical allodynia in rats by increasing the sensitivity to thermal and mechanical stimuli of hind paws that have been rendered hyperalgesic by unilateral sciatic nerve ligation. SR141716A did not alter the sensitivity of unlesioned paws to these stimuli. The data from this investigation *provided support for a role of the endocannabinoid system in the regulation of nociceptive thresholds in hyperalgesic but not non-hyperalgesic tissue* [**13**].

The same rodent neuropathy model was used to show that intrathecal injections of Δ9THC, a partial CBR agonist, were also effective in lengthening withdrawal latencies to thermal stimuli. The selective central cannabinoid receptor antagonist SR141716A, but not the generic opioid receptor antagonist naloxone, blocked the delta9-THC antinociception. It was stated that *the cannabinoid analgesic system might be superior to opioids in alleviating neuropathic pain syndromes* [**47**].

*Partial ligation of the rat sciatic nerve* produced similar changes to hind limb sensory stimulus thresholds like CCI, manifested as allodynia and hyperalgesia.

Bridges et al. (2001) [**48**] showed that WIN-55,212-2 reversed the signs of neuropathy at doses that did not generally alter sensory thresholds in the contra lateral unligated limb, and there was a dose-effect relationship. This effect was prevented by co-administration of the CB1 receptor antagonist SR141716a, but not by co-administration of the CB2 receptor antagonist SR144528. Administration of SR141716a alone had no affect on the observed allodynia and hyperalgesia. These results confirm the anterior hypotheses of Herzberg et al.

Systemic chronic treatment with WIN-55,212-2, CP-55,940 (a complete CBR agonist) or HU210 (a complete CB1R agonist) after nerve injury produced a dose-related reversal of mechanical hyperalgesia [**49**]. With higher doses of these cannabinoids, anti-nociceptive effects on contralateral withdrawal thresholds and side effects of catalepsy and sedation were also observed. Of these three CBs, WIN-55,2212-2 had the best side effect profile and was also reported to reverse mechanical allodynia and reduce thermal hyperalgesia. In addition, intrathecal injections of WIN-55,212-2, as well as local injections into hind paw tissue, were effective in reducing mechanical hyperalgesia.

In spite of these findings, other authors showed that the effect of intravenous administration of WIN-55,212-2 appeared to be (only) centrally mediated because administration of the drug directly to the ligated nerve did not suppress the heat-evoked neuronal activity (heat-evoked firing of spinal wide dynamic range neurons) at CCI rats [**50**]. *A neural basis for reports of potent suppression by cannabinoids of the abnormal sensory responses that resulted from nerve injury* was obtained.

The role of CB2 agonists in neuropathic pain was also explored. Tactile and thermal hypersensitivity produced by *spinal nerve ligation* (the ligation of both L6 and L5 spinal nerves) was dose-dependently inhibited by systemic administration of the CB2 receptor agonist AM1241 [**51**]. The analgesic action of AM1241 injected directly into the hind paw skin, has been demonstrated in both acute and inflammatory pain models but not so far in neuropathic animals [**52**,**53**,**54**,**55**].

In vivo experiments evaluated the effects of spinal administration of JWH-133, a CB2 receptor agonist, on mechanically evoked responses of neuropathic and sham-operated rats. Spinal JWH-133 attenuated mechanically evoked responses of spinal neurons in neuropathic, but not sham-operated rats. Data provided evidence that at the level of the spinal cord, CB2 receptors had inhibitory effects in neuropathic, but not sham-operated rats suggesting that *spinal CB2 may be an important analgesic target *[**56**].

Intrathecal administration of JWH133 significantly reversed partial sciatic nerve ligation-induced mechanical allodynia in mice at 0.5 h after administration. In contrast, systemic (intraperitoneal) or local (injected to the dorsal surface of the hind paw) administration of JWH133 was ineffective. Furthermore, the analgesic effects of intrathecal JWH133 were absent in cannabinoid CB2 receptor knockout mice. These results suggest that *the activation of central, but not peripheral, cannabinoid CB2 receptors play an important role in reducing mechanical allodynia* [**57**].

Intrathecal injection of CP-55940, a complete CBR agonist, was also effective in inhibiting tactile allodynia in this model, although treatment was associated with significant behavioural toxicity that could be alleviated by treatment with CB1 but not CB2 receptor antagonists [**58**].

Costa et al. 2004 investigated the effect of repeated treatment with the synthetic cannabinoid receptor agonist WIN-55,212-2 on neuropathic pain induced in rats by chronic constriction of the sciatic nerve. WIN-55,212-2, administered daily throughout the development of neuropathy, reversed the hyperalgesia, at a dose that had no effect on the nociceptive responses of either paw contralateral to the sciatic ligation or of animals subjected to sham surgery. At 14 days after injury, the levels of mediators known to be involved in neuropathic pain, such as prostaglandin E2, nitric oxide (NO) and the neuronal nitric oxide synthetase (NOS), were increased. Repeated treatment with WIN-55,212-2 abolished these increases [**59**].

Accumulating evidence suggests that cannabinoids can produce antinociception through peripheral mechanisms.

In another study [**60**], anesthetized rats received a mild heat injury to one hind paw and exhibited hyperalgesia as evidenced by lowered withdrawal latency to radiant heat and increased withdrawal frequency to a von Frey monofilament delivered to the injured hindpaw. WIN-55,212-2 attenuated both heat and mechanical hyperalgesia dose-dependently.

The CB1 receptor antagonist AM 251 co-injected with WIN-55,212-2 attenuated the antihyperalgesic effects of WIN- 55,212-2. The CB2 receptor antagonist AM 63 co-injected with WIN-55,212-2 attenuated only early effects of WIN-55,212-2.

These data demonstrate that cannabinoids primarily activate peripheral CB1 receptors to attenuate hyperalgesia.

WIN-55,212-2 perineural continuously delivered to the site of a partial ligation injury to the sciatic nerve reduced hypersensitivity to stimuli applied to the injured limb several days after injury. When delivered to the contralateral side to injury, WIN 55,212-2 did not significantly affect nerve injury-associated hypersensitivity. Co-perineural application of a CB1R antagonist SR-141716a and WIN-55,212-2 prevented the effects of WIN-55,212-2 on hypersensitivity. Co-application of CB2R antagonist SR-144528 reversed WIN-55,212-2's effect on mechanical hypersensitivity on day 2 only.

These data support a peripheral antihyperalgesic effect of WIN- 55,212-2 when delivered directly to the site of a nerve injury at systemically inactive doses [**61**].

Intraplantar pretreatment with WIN-55,212-2 before capsaicin produced a dose-dependent attenuation of hyperalgesia to heat. SR141716A co-injected with WIN-55,212-2 partially attenuated the effects of WIN- 55,212-2 on hyperalgesia to heat. Intraplantar injection of the highest dose of WIN-55,212-2 did not interfere with the development of hyperalgesia following capsaicin injection into the contralateral paw.

These data show that cannabinoids possess antihyperalgesic properties at doses that alone do not produce antinociception, and are capable of acting at both spinal and peripheral sites [**62**].

## Conclusions and therapeutic perspectives

The efficacy of cannabinoid receptors agonists on neuropathic pain is supported by considerable preclinical and clinical evidence and there are anecdotal reports to suggest that smoking *cannabis* may relieve the pain and spasticity in multiple sclerosis sufferers. Limited clinical trials using various forms of THC, the major active component of cannabis, have shown it to have analgesic activity in some forms of neuropathic pain [**63**].

Neural basis for reports of potent suppression by cannabinoids of the abnormal sensory responses that resulted from nerve injury was presented. Cannabinoid receptors, especially CB1 receptors, were found in the pathways of nociceptive sensations transmission and in key regions of pain control (periaqueductal gray, rostroventromedial medulla). Their stimulation produced analgesia.

Studies of pain pharmacology showed a role for the endocannabinoid system in the regulation of nociceptive thresholds in hyperalgesic but not non-hyperalgesic peripheric tissues. Thus, the cannabinoid analgesic system might be superior to opioids in alleviating neuropathic pain syndromes. 

Recent findings showed also that spinal CB2 receptors might be an important analgesic target in neuropathic pain although they probably are not found in neurons or synapses.

Accumulating evidence suggested that cannabinoids could produce antinociception through peripheral mechanisms.

In order to develop new analgesic drugs that influence the cannabinoid system, drugs active in neurophatic pain and not only, it’s obvious to take into consideration 3 directions:

- the development of CB1 receptors agonist drugs which do not pass the blood brain barrier. In this way, the use of liposoluble compounds active on CB1 receptors can be avoided and thus negative psychotropic effects mentioned above;

- the development of CB2 receptors agonist drugs which do not have effects on CB1 receptors;

- the possible use of some modulators of endocannabinoids metabolization (this issue was not detailed in the present review, but for further in formations reference 17 can be consulted).
